# Novel Variant Expands the Clinical Spectrum of CUX2-Associated Developmental and Epileptic Encephalopathies

**DOI:** 10.3389/fgene.2022.808181

**Published:** 2022-07-01

**Authors:** Feng Zhang, Fuwei Li, Fujian Chen, Jinrong Huang, Qiong Luo, Xilong Du, Jiapeng Zhou, Weiyue Gu, Kaishou Xu

**Affiliations:** ^1^ Ganzhou Women and Children’s Health Care Hospital, Ganzhou, China; ^2^ Beijing Chigene Translational Medical Research Center Co. Ltd., Beijing, China; ^3^ Guangzhou Women and Children’s Medical Center, Guangzhou Medical University, Guangzhou, China

**Keywords:** trio-whole exome sequencing, developmental and epileptic encephalopathy 67, CUX2, levetiracetam treatment, CUX2 clinical phenotype

## Abstract

Developmental and epileptic encephalopathies (DEE) caused by heterozygous deleterious variants in Cut Like Homeobox2 (*CUX2*) is rare. To the best of our knowledge the only variant associated with a phenotype in this gene is the *de novo* missense variant c.1768G > A, p.Glu590Lys; however, further additional research is needed to characterize the relationship between disease and variants in this gene. In this study, we reported a patient from a non-consanguineous Chinese family presenting with epilepsy, developmental delay, and speech delay. Additionally, the patient responded well to levetiracetam, and at his last follow-up (5.5 years old), he had discontinued antiepileptic drug treatment and remained seizure-free for 6 months. To identify possible causative variants, trio-whole exome sequencing was performed. We identified a novel *de novo* missense *CUX2* c.2834C > T, p. Thr945Met variant in the patient. Based on clinical and genetics information associated with the bioinformatics analyses, we hypothesized that this variant was the cause of the reported phenotype. AlphaFold and SWISS-MODEL homology modeling servers were used to predict the three-dimensional (3D) structure of CUX2 protein. Predictions based on the 3D-structure modeling indicated that the p.Thr945Met substitution was likely to alter the DNA-binding specificities and affect protein function. On the basis of clinical characteristics and genetic analysis, we presented one case diagnosed with DEE67. Our finding expanded the clinical and molecular spectrum of *CUX2* variants.

## Introduction

Developmental and epileptic encephalopathies (DEE) is characterized by onset in infancy/early childhood with refractory seizures, delayed psychomotor development or/and developmental regression. DEE is clinically and genetically heterogeneous, with over 50 genes known to be causative (Specchio and Curatolo, 2021). However, the genotype still has a limited effect on the phenotype. The Cut Like Homeobox 2 (*CUX2*) gene, which encodes a 1426 amino-acids transcription factor, plays an important role in the control of neuronal proliferation and differentiation in the brain (Quaggin et al., 1996). Recently, variants in *CUX2* were found to be associated with developmental and epileptic encephalopathy 67 (DEE67, OMIM: 618141), an autosomal dominant disorder that typically manifests in infancy and is characterized by refractory seizures, global developmental delay with impaired motor and intellectual development, movement disorders, speech delay, and stereotypic or autistic behavior. To date, only 10 patients carrying recurrent *de novo* heterozygous Glu590-to-Lys (E590K) variant in the CUX2 gene have been reported (Barington et al., 2018; Chatron et al., 2018).

Here, we reported a 5.5 years old boy with epilepsy, developmental delay and speech delay. Trio (parents-proband) whole-exome sequence (WES) analysis revealed a novel *de novo* pathogenic missense variant c.2834C > T (p.Thr945Met) in the CUX2 gene. Our findings expand the genetic and phenotypic spectrum of DEE67 and contribute to our understanding of DEE on a genetic level.

## Material and Methods

### Patients

The proband was a 5.5-year-old boy, the third child of healthy non-consanguineous parents. He had two unaffected female siblings. The patient visited the hospital after experiencing seizures. A detailed clinical history, including a description of epilepsy, a detailed clinical, and a neurological examination was carried out. The study was approved by the Ethics Committee of Ganzhou Women and Children’s Health Care Hospital.

### Variant Analysis

After obtaining informed consent, samples were taken from the proband and his parents. The whole-exome capture was carried out (xGen Exome Research Panel v1.0, IDT, IA, United States) according to the manufacturer’s protocol. High-throughput sequencing was performed using Illumina NovaSeq 6000 series sequencer (PE150), and at least 99% of the target sequence was sequenced. The sequencing process was performed by the Chigene Translational Medicine Research Center Co., Ltd., 100875, Beijing.

After eliminating adapters and low-quality reads, clean data were obtained and aligned to the Human genome (Hg19/GRC37) using the Burrows-Wheeler Alignment (BWA). The Genome Analysis Toolkit (GATK) was used to identify single nucleotide variants (SNVs) and insertion/deletion (InDel) variants. The Exome Aggregation Consortium ExAC was used to determine the allele frequencies of variants. Pathogenicity of nonsynonymous variants was predicted using the SIFT, Provean, REVEL, varianttaster, PolyPhen, and CADD tools. SNVs/indels were classified according to the standards and guidelines of the American College of Medical Genetics and Genomics (ACMG) and the Association for Molecular Pathology (AMP) (Richards et al., 2015).

Variants were confirmed by Polymerase Chain Reaction (PCR) analysis, followed by Sanger sequencing. *CUX2* was amplified using the following primers: forward primer (5′-CAG​CCT​GGT​ACA​AGT​CCC​AAC-3′) and reverse primer (5′-CAG​ACT​TAT​CCG​CTG​GTC​CC-3′). DNA sequencing was performed using the 3730xl DNA Analyzer (Applied Biosystems, United States). The rare variant identified has been submitted to the ClinVar database (Accession number: SCV001946791) (https://www.ncbi.nlm.nih.gov/clinvar/variation/1296981).

Multiple protein sequence alignments were performed using MEGA X, and the modeling of the 3D structure was analyzed and visualized using Swiss-PdbViewer v4.1.

## Results

### Clinical Features

The proband (II:1) had the onset of febrile seizure at the age of 2 years with unconsciousness, up-rolling of the eyes, facial twitching, limb stretch, and no visible limb shaking; 6 months later, a second febrile seizure occurred, and 3 months later a third febrile seizure occurred. Each seizure lasted 3 min and was followed by spontaneous remission. At 2 years and 9 months the medical team started levetiracetam (40 mg/kg/day) and patient did not present any other febrile seizure. After 2 years without clinical or electrographic seizures, the boy has discontinued levetiracetam and remained seizure-free until the age of five. At 2 years and 9 months, the electroencephalograph (EEG) revealed a somewhat slower background activity and a small number of bilateral spikes and spike-wave discharges in the central, parietal, and posterior temporal regions during sleep, but subsequent magnetic resonance imaging (1.5T MRI) was normal. EEG performed at the age of 5 years without levetiracetam was normal. He was able to walk alone at 18 months. At the age of 2 years and 9 months, the patient was diagnosed with global developmental delay and he was not able to form three word sentences and just could pronounce few words with correct meaning. The results of the Gesell Development Diagnosis Scale (GDDS) revealed a total development quotient (DQ) of 56 (mild developmental delay). Then the patient has received home-based rehabilitation for 3 years. Now he is 5.5 years in kindergarten and the DQ score has been improved to 75 (borderline deficiency).

### Variant Identification and Analysis

**FIGURE 1:**
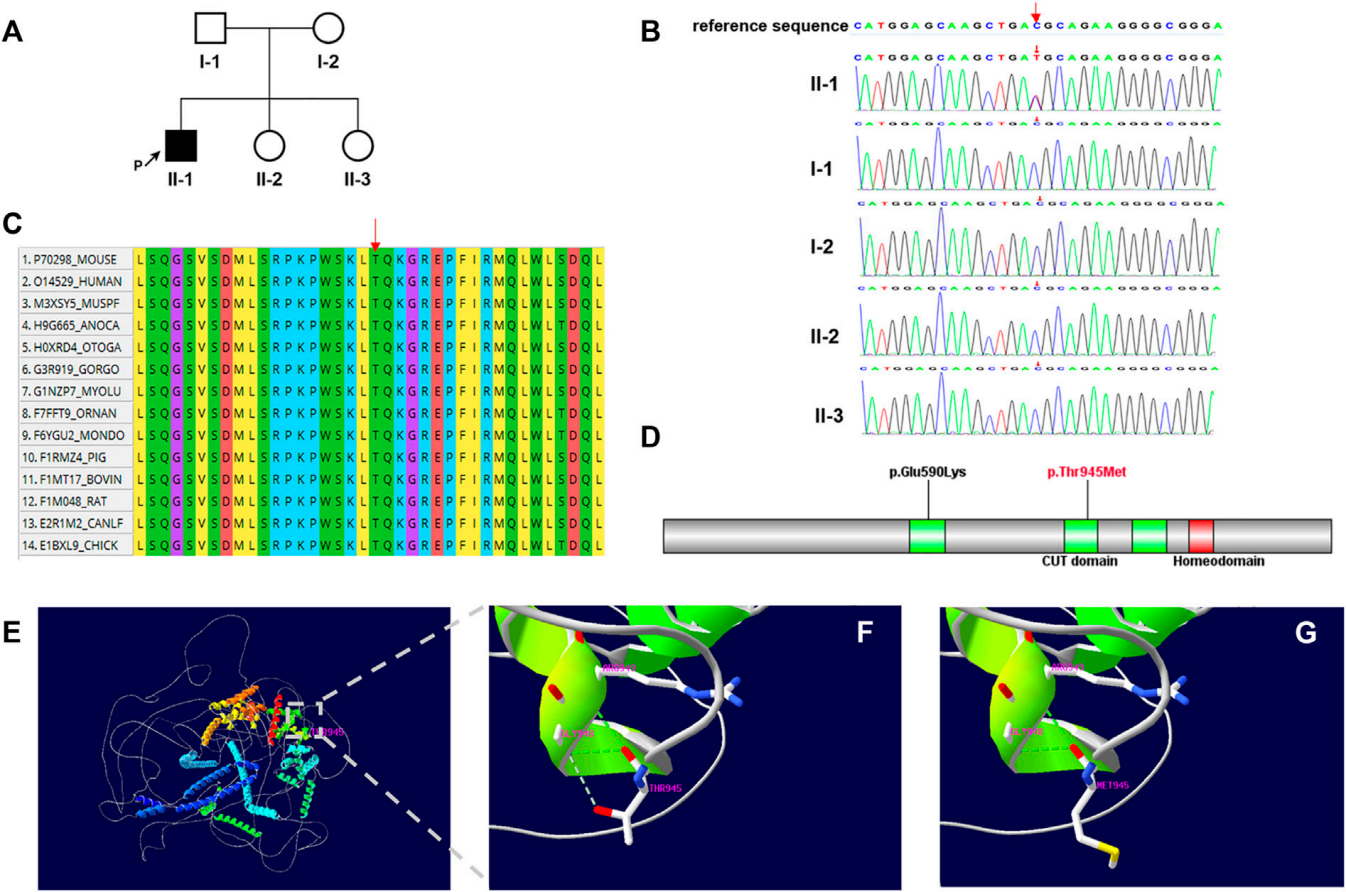
**(A)** Family pedigree. The black arrow indicates the proband. **(B)** Sanger sequencing of the patient and family members confirmed the *de novo* variant c.2834C > T. **(C)** Multiple-sequence alignment of CUX2 protein among species. Red arrow shows the Thr945 site. **(D)** Domain structure of CUX2 protein (CUT domain, green; Homeodomain, red). Localization of amino acid changes p.Glu590Lys and p.Thr945Met is indicated. **(E–G)** Structural model of the CUX2 protein. Ribbon representations show details of the second CUT domain for wild-type (p.Thr945) **(F)** and mutated (p.Thr945Met) **(G)** CUX2. Noted loss of the weaker hydrogen bond between Thr945 and Gly948. Green dashed lines: hydrogen bonds; grey dashed lines: weaker hydrogen bonds.

Trio-WES was performed and a heterozygous *de novo* missense variant (NM_015267.4: c.2834C > T, p.Thr945Met), in the CUX2 gene was identified in the proband. This c.2834C > T variant was not identified in the 1000 genome project or ExAC. Numerous computational tools, including PolyPhen (probably damaging, 1.0), Provean (deleterious, −4.16), SIFT (damaging, 0.0), mutationtaster (disease_causing, 1), and REVEL (deleterious, 0.582) suggested that the variant was deleterious and had a CADD score of 26.9. The *de novo* missense variant was confirmed by Sanger sequencing, the proband’s parents and healthy sisters did not carry the variant (Figures 1A,B). Based on these findings, the variant was classified as “Likely pathogenic” by ACMG/AMP guidelines (PS2, PM2, PP2, and PP3) (Richards et al., 2015; Harrison et al., 2019).

Multiple sequence alignment of the protein shows that the Thr-945 residue is highly conserved across species (Figure 1C). This variant locates in the second CUT domain of the human homeobox protein CUX2 (Cut-like2) (Figure 1D) and results in a threonine to methionine amino acid substitution. By substituting a polar and uncharged residue with a non-polar and hydrophobic amino acid, this variant may further affect the formation of a weaker hydrogen bond between Thr 945and Gly948 (Figures 1E–G).

## Discussion

In this study, we performed trio-whole exome sequencing and characterized a novel missense variant c.2834C > T (p.Thr945Met) in *CUX2* in a Chinese male patient with DEE. In previous studies, all known patients (ten unrelated probands with DEE) presented with a recurrent *de novo* missense variant, c.1768G > A (p.Glu590Lys) (Table 1) (Rauch et al., 2012; Allen et al., 2013; Geisheker et al., 2017; Barington et al., 2018; Chatron et al., 2018). The ten patients presented with a variety of epilepsy forms, including myoclonic seizures (3), absence seizures (2), focal, tonic, atonic, and generalized tonic-clonic. The age of onset ranged from 2 months to 9 years. EEG was performed in 9 of 10 patients. All participants had abnormal EEG findings, and 5 individuals presented with generalized spike-wave (GSW) or polyspike-wave (GPSW) patterns. Brain MRI was performed in 9 of 10 patients and was unremarkable in 6 of 9 patients. Cerebellar atrophy, hippocampal asymmetry, thin posterior corpus callosum were observed in some patients. In our case, the EEG revealed bi-parieto-temporal discharges while the MRI was normal. All patients had developmental delays, in addition to a variety of other variable features such as nonverbal (7), movement disorders (6), and autistic features (3). Our patient presented with febrile seizure when 2 years old. Besides, he has developmental delay.

**TABLE 1:** Clinical features of individuals with the CUX2 variant.

Individual	*De novo* variant	Age	Sex	Type of seizure	Age at seizure onset	Seizure outcome	EEG features	MRI	ID	ASD	Non verbal	Movement disorder	Other features
1 (this study)	c.2834C > T, p.Thr945Met	5.5 years	M	Febrile seizure	2 years	Seizure free	Bi-parieto-temporal discharges	Normal	NA	No	No	No	Speech delay
2 Chatron et al. (2018)	c.1768G > A, p.Glu590Lys	19 years	M	Myoclonic seizures and right occipital seizures with apnea	7 months	Refractory	GSW, sometimes with myoclonic seizures. Right occipital seizure recorded	Normal	Profound	NA	Yes	Yes	Hypotonic at 1 year, then spastic tetraparesis from 12 years with loss of ambulation.
3 Chatron et al. (2018)	c.1768G > A, p.Glu590Lys	21 years	M	Atypical absences with myoclonus	6 months	Refractory	3–4 Hz GSW, GPSW	Cerebellar atrophy	Severe	NA	Yes	NA	No eye contact, inappropriate laughter episodes
4 Chatron et al. (2018)	c.1768G > A, p.Glu590Lys	9 years	F	Myoclonic	6 months	Refractory	GSW, GPSW	Hippocampal asymmetry	Severe	NA	Yes	NA	Decreased reflexes, ataxic gait
5 Chatron et al. (2018)	c.1768G > A, p.Glu590Lys	14 years	F	Absence seizures	12 months	Refractory	3 Hz GSW	Normal	Severe	NA	NA	Yes	NA
6 Chatron et al. (2018)	c.1768G > A, p.Glu590Lys	16 years	M	Myoclonic seizures	5 months	Refractory	GSW, GPSW	Normal	Severe	Yes	Yes	NA	NA
7 Chatron et al. (2018)	c.1768G > A, p.Glu590Lys	6 months	M	Generalized tonic-clonic seizures, atypical absences with apnea and myoclonus, right hemiclonic seizures	2 months	Refractory	Multifocal discharges, mainly independent bi-parieto-temporal	Normal	NA	NA	NA	Yes	NA
8 Barington et al. (2018)	c.1768G > A, p.Glu590Lys	17 years	F	Generalized and myoclonic seizures.	12 months	Seizure free	NA	NA	Severe	Yes	Yes	Yes	Sialorrhea, chronic constipation
9 Rauch et al. (2012)	c.1768G > A, p.Glu590Lys	8 years	M	Focal spasms	6 months	Seizure free	Hypsarrhythmia	Normal	Severe	NA	Yes	Yes	NA
10 Allen et al. (2013)	c.1768G > A ,p.Glu590Lys	12 years	M	Myoclonic seizures	2 months	Refractory	Left temporal spikes/sharp waves	Normal	Severe	NA	NA	Yes	Ataxic gait, inappropriate laughter episodes
11 Geisheker et al. (2017)	c.1768G > A, p.Glu590Lys	14 years	M	Absence seizures	9 years	Seizure free	Left fronto-central slowing	Thin posterior corpus callosum	Severe	Yes	Yes	NA	NA

EEG, electroencephalograph; MRI, magnetic resonance imaging; ID, intellectual disability; ASD, autistic spectrum disorder; M, male; GSW, generalized spike-wave; NA, not available; GPSW, generalized polyspike-wave; F, female.

The majority of patients with the p.Glu590Lys variant failed to respond to multiple antiepileptic drugs, including valproate, carbamazepine, clobazam, levetiracetam, and lamotrigine. Only three patients achieved seizure-free status while using valproate or a combination of valproate and lamotrigine. In our case, seizures were controlled after treatment with levetiracetam (Barington et al., 2018; Chatron et al., 2018). At the last follow-up, he had discontinued antiepileptic drug treatment (at age of 5 years) and remained seizure-free for 6 months. Taken together, our patient had a less severe phenotype than previously reported in patients carrying the p.Glu590Lys variant, although the relationship between the reported variant and the described phenotype requires more case reports to be established.

The CUX2 protein consists of three CUT domains and a homeodomain; which are important for DNA binding (Gingras et al., 2005). Additionally, the sequences of all four DNA-binding domains are highly conserved (Quaggin et al., 1996). The CUT domain 1 is located between amino acids 549 and 627, while the CUT domain 2 is located between amino acids 892 and 967 in CUX2. The previously reported variant p.Glu590Lys, which is located at the CUT domain 1, destabilized the effect in-silico analyses and interfered with DNA binding (Chatron et al., 2018). In this study, we modeled the 3D structure of the wild-type CUX2 protein (1–1486) and examined the potential functional impact of the p.Thr945Met variant (Figures 1E–G). Firstly, the variant alters the charge at residue 945 (polar to non-polar) (CUPSAT: ΔΔG = −6.21 kcal/mol, I-mutant3.0: ΔΔG = −0.37 kcal/mol), potentially disrupting the structure and affecting inter and intramolecular interactions (Chatron et al., 2018). Additionally, the Thr945Met variant affects a highly-conserved threonine residue in the CUT domain 2, which could result in structural changes and alter DNA-binding specificities (Gingras et al., 2005). However, additional molecular functional studies will be required to confirm the potential mechanism of pathogenic variants.

In summary, patients with *de novo* CUX2 variants have developmental and epileptic encephalopathies characterized by developmental delay, speech delay, movement disorders, and autistic behavior. Our findings have further expanded the clinical and molecular spectrum of DEE67.

## Data Availability

The datasets presented in this study can be found in online repositories. The names of the repository/repositories and accession number(s) can be found below: https://www.ncbi.nlm.nih.gov/, SCV001946791.
